# Identification and Characterization of Circulating MicroRNAs as Novel Biomarkers in Dogs With Heart Diseases

**DOI:** 10.3389/fvets.2021.729929

**Published:** 2021-10-11

**Authors:** Woong-Bin Ro, Min-Hee Kang, Doo-Won Song, Heyong-Seok Kim, Ga-Won Lee, Hee-Myung Park

**Affiliations:** Department of Veterinary Internal Medicine, College of Veterinary Medicine, Konkuk University, Seoul, South Korea

**Keywords:** microRNA, circulating, dog, canine, heart disease, cardiac hypertrophy, novel biomarker, therapeutic target

## Abstract

**Background:** Previous studies in humans have confirmed dysregulations of circulating microRNAs (miRNAs) in patients with various cardiovascular diseases. However, studies on circulating miRNAs in dogs with various heart diseases are limited in number. This study aimed to identify significantly dysregulated circulating miRNAs and characterize them as novel biomarkers in dogs with heart diseases.

**Materials and Methods:** Circulating levels of 11 miRNAs were investigated in serum samples of 82 dogs (72 with heart diseases and 10 healthy dogs) using quantitative reverse transcription-polymerase chain reaction. The results were correlated to clinical data including echocardiographic results and N-terminal pro B-type natriuretic peptide (NT-proBNP) levels.

**Results:** Upregulation of cfa-miR-130b was observed in dogs with myxomatous mitral valve degeneration (MMVD) stage B, patent ductus arteriosus, and pulmonic stenosis. In dogs with MMVD stage B, cfa-miR-130b was upregulated and correlated with clinical indices. In receiver operating characteristic (ROC) analysis, cfa-miR-130b accurately distinguished dogs with diseases from healthy dogs. We also observed that cfa-miR-375 and cfa-let-7b were upregulated in dogs with concentric cardiac hypertrophy. The cfa-miR-375 was correlated with concentric hypertrophy indices and was an accurate indicator of concentric hypertrophy in ROC analysis.

**Conclusions:** The miRNAs identified in this study may be used as novel biomarkers and possible candidates for therapeutic targets in various canine heart diseases.

## Introduction

MicroRNAs (miRNA) are small, non-coding single-stranded RNAs consisting of 19–24 nucleotides, which form complementary pairs with target mRNAs to inhibit and regulate their expression through translation inhibition or degradation (1). Previous studies have shown that miRNAs are involved in cardiac development and play crucial roles in pathological processes of cardiovascular diseases (2, 3). In humans, altered expressions of miRNAs were reported in various heart diseases (4–6), and circulating miRNAs are increasingly investigated as novel biomarkers in heart diseases because of its stability in peripheral blood (7). In addition, since cardiac hypertrophy is one of the most important pathological response in heart diseases, miRNAs related to cardiac hypertrophy are considered as promising therapeutic targets for various cardiovascular diseases (8, 9).

However, little is known about expressions and role of circulating miRNAs in dogs with naturally occurring heart diseases. To date, several studies have reported dysregulation of circulating miRNAs in dogs with myxomatous mitral valve degeneration (MMVD) (10–13), and no significant change of circulating miRNAs was reported in a previous study in dogs with dilated cardiomyopathy (DCM) (14). However, circulating miRNAs in heart diseases other than MMVD and DCM, such as patent ductus arteriosus (PDA) and pulmonic stenosis (PS), have not been studied in dogs. Moreover, there have been no studies in dogs that have evaluated and characterized the dysregulated miRNAs as novel biomarkers or candidate for therapeutic targets through further analysis with clinical data.

This study aimed to identify significantly dysregulated circulating miRNAs and evaluate them as novel biomarkers in dogs with various heart diseases, and also investigate and characterize circulating miRNAs associated with specific cardiac hypertrophy type.

## Materials and Methods

### Study Design

This study is a retrospective study. In this study, serum levels of 11 candidate miRNAs associated with cardiac hypertrophy in a previous study in dogs (cfa-miR-130b, cfa-miR-346, cfa-let-7b, cfa-miR-30c, cfa-miR-30d, cfa-miR-19b, cfa-miR-425, cfa-let-7g, cfa-miR-151, cfa-miR-375, and cfa-miR-505) were investigated in dogs with various heart diseases using quantitative reverse transcription-polymerase chain reaction (qRT-PCR) (15). The results of qRT-PCR and clinical data including medical records, echocardiographic results, and N-terminal pro B-type natriuretic peptide (NT-proBNP) levels were analyzed together. Bioinformatics analysis was performed on miRNAs with significant results to identify functions and pathways of the target genes.

**Figure 1:**
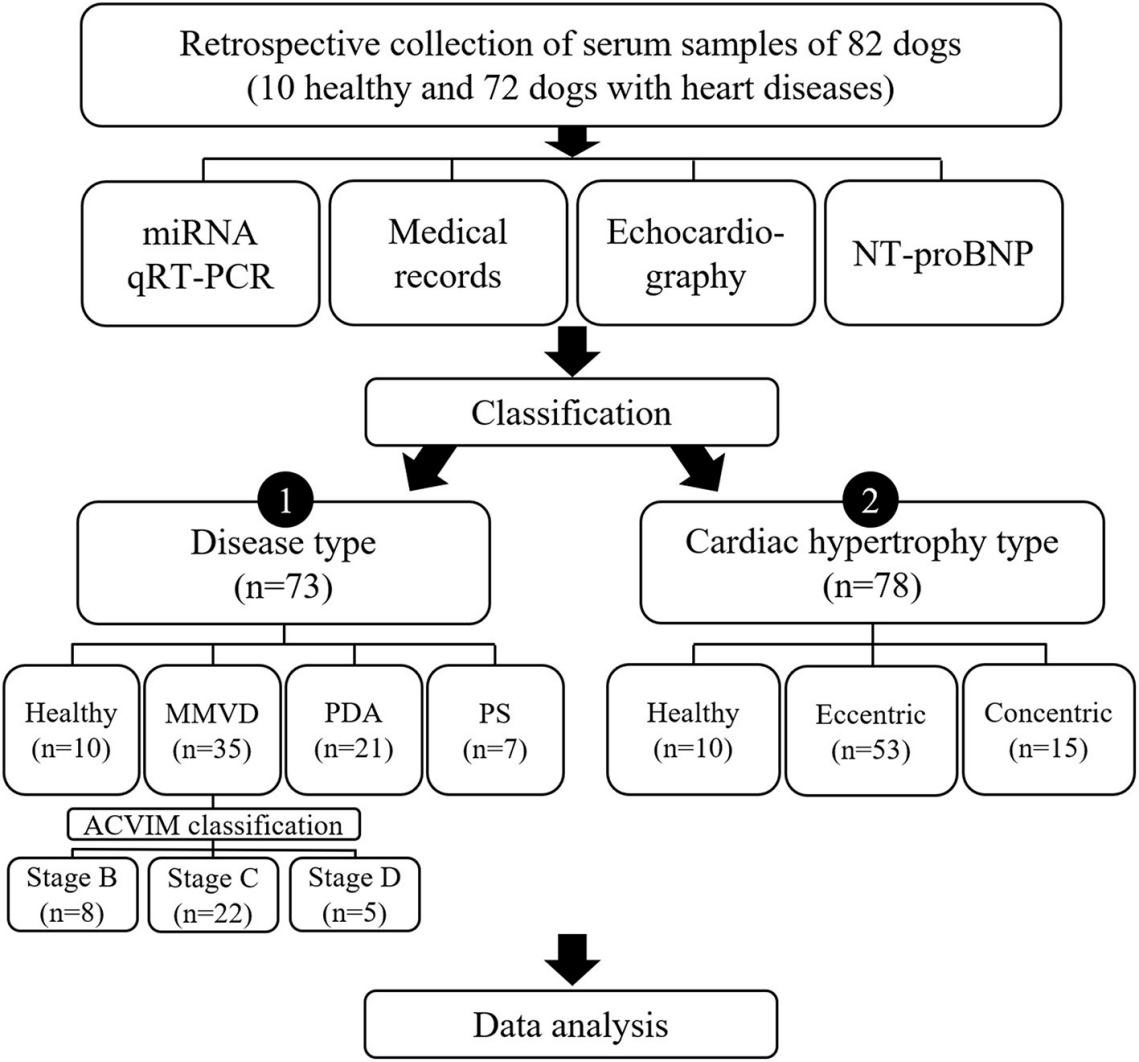
Study design. Results of qRT-PCR on 11 miRNAs, medical records, echocardiography, and NT-proBNP measurement in 82 dogs were analyzed in this study. To investigate different factors related to expression of miRNAs, the dogs included in this study were sub-grouped by two different classifications, classification by disease type and classification by cardiac hypertrophy type, and each classification was analyzed independently. ACVIM, American College of Veterinary Internal Medicine; miRNA, microRNA; MMVD, myxomatous mitral valve degeneration; NT-proBNP, N-terminal pro B-type natriuretic peptide; PDA, patent ductus arteriosus; PS, pulmonic stenosis; qRT-PCR, quantitative reverse transcription-polymerase chain reaction.

To investigate different factors related to expression of miRNAs, the dogs included in this study were sub-grouped by two different classifications, classification by disease type and classification by cardiac hypertrophy type, and each classification was analyzed independently (Figure 1).

### Sample Collection

Stored serum samples of 10 healthy dogs from a previous study were utilized in this study. The previous study was conducted under the supervision of Korea Institute for Advancement of Technology (KIAT) for Regional Specialized Industry Development Program (R&D, R0006046) and approved by KBNP Institutional Animal Care and Use Committee (KBNP 18-01-01). All healthy dogs were confirmed to be healthy in physical examination, complete blood count, serum chemistry, and urinalysis. Stored serum samples of 72 dogs with heart diseases were also retrospectively collected from the Veterinary Medical Teaching Hospital of Konkuk University between July 2014 and January 2019. Informed owner consent was obtained.

The inclusion criteria for dogs with heart diseases were as follows: (1) acquired or congenital heart diseases or (2) cardiac hypertrophy by non-cardiac cause, such as hyperadrenocorticism (HAC). Exclusion criteria were systemic disorders other than HAC. Dogs with heart diseases and concurrent HAC were included in the study because HAC is known to induce cardiac hypertrophy in dogs (16). The diagnoses of MMVD, patent ductus arteriosus (PDA), pulmonic stenosis (PS), and HAC followed the proposed guidelines in dogs, as previously described (17–20). Dogs with MMVD were classified according to the American College of Veterinary Internal Medicine (ACVIM) consensus guidelines as follows (20): stage B included MMVD dogs that had not yet developed clinical signs due to heart failure [stage B1 had no cardiac remodeling or had remodeling not enough to meet the criteria for stage B2, while stage B2 had cardiac enlargement to meet the criteria of murmur intensity ≥ 3/6; ratio of left atrial to aortic diameter (LA/Ao) ≥ 1.7; normalized value of end-diastolic LV internal dimension (LVIDdN) ≥ 1.7; vertebral heart score (VHS) > 10.5], stage C included dogs with present or past clinical signs due to heart failure caused by MMVD, and stage D included dogs with end-stage MMVD that were refractory to standard treatment.

### Case Classification

In the classification by disease type, dogs were classified by their diagnosis of heart disease to identify the changes of miRNA in various heart diseases. Dogs with concurrent HAC, those with more than two congenital heart diseases (e.g., tetralogy of Fallot), or those not diagnosed with heart disease were excluded from this classification.

In the classification by cardiac hypertrophy type, dogs were classified into eccentric hypertrophy or concentric hypertrophy groups. Eccentric hypertrophy group was defined as dogs with volume overload heart diseases (e.g., MMVD and PDA) with evidence of increased left ventricle (LV) or left atrium (LA) cavity: LA/Ao > 1.13 or LVIDdN > 1.73 or normalized value of end-systolic LV internal dimension (LVIDsN) > 1.14 (21, 22). Concentric hypertrophy group was defined as dogs with pressure overload heart diseases (e.g., PS and tetralogy of Fallot), or dogs with concentric hypertrophy by non-cardiac cause (e.g., hypertension, HAC) (16, 23, 24) with evidence of increased LV wall thickness: normalized value of end-diastolic interventricular septal thickness (IVSdN) > 0.52 or normalized value of end-diastolic LV free wall thickness (LVPWdN) > 0.53 (22).

### Clinical Data and NT-proBNP Measurement

Clinical data including breed, age, sex, body surface area (BSA), heart rate (HR), blood pressure, and cardiovascular medication history were retrieved from medical records. Serum concentration of NT-proBNP was measured by enzyme-linked immunosorbent assay (IDEXX Laboratories Inc., Westbrook, ME, USA).

### Echocardiographic Evaluation

Echocardiographic data were obtained from previous medical records. Examinations were performed on conscious unsedated dogs. Standard two-dimensional, spectral, and tissue Doppler echocardiographic examinations were performed in the left and right lateral recumbency with continuous monitoring of electrocardiography (ECG). A high-quality echocardiograph (EPIQ 7 ultrasound system, Philips Medical Systems, Andover, MA, USA) was used.

M-mode measurements of LV were acquired at the chordae tendineae level from the standard right parasternal short axis view. These measurements included IVSd, LVPWd, LVIDs, LVIDd, and fractional shortening (FS). The LV measurements were normalized using body weight (IVSdN, LVPWdN, LVIDsN, and LVIDdN) according to the results of a previous study on regression analysis (22). LV hypertrophy was evaluated by the normalized measurements based on the 95% prediction interval for normal range (22). Calculations of LV volumes (end-diastolic and end-systolic volumes) and ejection fraction (EF) were derived from Teichholz's formula. The end-diastolic volume index (EDVI) and end-systolic volume index (ESVI) were calculated as the following formula: EDVI or ESVI = (end-diastolic volume or end-systolic volume)/BSA. The LA/Ao was calculated from the right parasternal short axis view by using B-mode. Relative wall thickness (RWT) was calculated as an indicator of LV hypertrophy. RWT was measured as the following formula: RWT = (IVSd + LVPWd)/LVIDd (23). The LV mass (LVM) as well as LV mass index (LVMI) were calculated for additional assessment of LV remodeling. The LVM was calculated as the following formula: LVM (g) = 0.8 × (1.04 × (LVIDd + LVPWd + IVSd)^3^ – (LVIDd)^3^) + 0.6. The LVMI was calculated as the following formula: LVMI = LVM/BSA (23). Trans-mitral flow was obtained by pulsed-wave Doppler from the left apical four-chamber view to measure the peak velocities of early diastolic wave (E) and late diastolic wave (A). The mitral annular motion velocity of the interventricular septum was obtained from the left apical four-chamber view by pulsed wave tissue Doppler. The peak velocity of the mitral annular motion in systole (S′), early diastole (E′), and late diastole (A′) were measured and the E/E' ratio was calculated. Pulmonary hypertension was tentatively diagnosed when peak tricuspid regurgitation flow gradient was confirmed to be 30–50 mmHg (mild), 50–75 mmHg (moderate), or more than 75 mmHg (severe) (25–27).

### RNA Preparation

RNA was extracted from serum samples using the miRNeasy Serum/Plasma Kit (Qiagen, Hilden, Germany) according to the manufacturer′s protocol. Briefly, 1 ml of QIAzol Lysis Reagent was mixed with 200 μl of serum. Following incubation at room temperature (RT) for 5 min, 3.5 μl of cel-miR-39 working solution (1.6 × 10^8^ copies/μl) was added as an exogenous spike-in to the lysate. RNA precipitation was carried out with 900 μl of 100% ethanol and 200 μl of chloroform in two separate steps. Subsequently, 700 μl of the sample was added to the RNeasy MinElute spin column and was centrifuged at 11,000 *g* and RT for 15 s. This was followed by washing of the columns with 500 μl of RPE buffer and 700 μl of RWT, centrifugation at 11,000 *g* and RT for 15 s, and precipitation of RNA with 500 μl of 80% ethanol, consecutively. RNAs were eluted from the columns with 14 μl of RNase-free water.

RNA quantity and integrity were evaluated with nanodrop 1000 Spectrophotometer (Thermo Scientific, Madison, WI, USA), Quant-IT microRNA assay kit by QuantusTM Fluorometer (Promega, Madison, WI, USA), and Agilent 2100 Bioanalyzer (Agilent Technologies, Palo Alto, CA, USA). No samples were excluded by low RNA quality.

### Quantitative Reverse Transcription-Polymerase Chain Reaction of miRNAs

Extracted RNA (5 μl) was used for cDNA synthesis using miScript II RT Kit (Qiagen), miScript Reverse Transcriptase Mix, 5 × miScript HiSpec Buffer, and 10 × miScript Nucleics Mix. The primers used in this study are shown in Supplementary Table 1. The mixture was incubated for 60 min at 37°C and 5 min at 95°C to inactivate the miScript Reverse transcriptase mix and placed on ice. The cDNA was diluted in RNase-free water (10 μl of cDNA obtained above was mixed with 40 μl of water). The diluted cDNA (5 μl) was preamplified using miScript PreAmp PCR Kit (Qiagen), miScript PreAMP Primer Mix, 5 × miScript PreAMP Buffer, HotstarTaq DNA Polymerase, and miScript PreAMP Universal Primer. Cycling conditions were 95°C for 15 min and 12 cycles of 94°C for 30 s and 60°C for 3 min on an ABI 9700 Thermal Cycler (Thermo Fisher Scientific, Waltham, MA, USA). The PreAmp Product was diluted in RNase-free water (25 μl of cDNA mixed with 475 μl of water).

miScript miRNA PCR array enabled and SYBR Green-based real-time PCR analysis was carried out using QuantStudio 12k Flex PCR system (Applied Biosystems, Darmstadt, Germany). In a 20 μl reaction, 1 μl of preamplified product was mixed with 5 μl of 2 × QuantiTect SYBR Green PCR Master Mix, 1 μl of 10 × miScript Universal Primer, and 1 μl of 10 × miScript Primer Assay. qRT-PCR was performed at 95°C for 15 min; 40 cycles of 94°C for 15 s; 55°C for 30 s; and 70°C for 30 s.

The qRT-PCR assays were done in triplicate with exogenous synthetic spiked-in miRNA across all the samples. Mean threshold cycles (Ct) for the synthetic miRNA and all samples were calculated. Seven endogenous and exogenous genes (SNORD61, SNORD68, SNORD95, SNORD72, SNORD96A, RNU6_2, and cel-miR-39) were selected as candidates for reference gene. In order to select the most suitable gene for internal reference, statistical analysis was performed using NormFinder software (28). According to the statistical algorithm, small nuclear RNA SNORD61 was selected as the most stabilized internal reference miRNA to normalize the cDNA levels of the samples. The Ct values obtained for each miRNA were normalized to the respective SNORD61 Ct value to obtain normalized Ct (ΔCt) values, which were subsequently used for statistical analysis. The ΔΔCt method was used to calculate fold change (FC) (2^−ΔΔCt^) relative to the healthy group.

### Statistical Analysis

The ΔCt values were used for statistical analysis. All data were expressed as mean ± standard deviation. Normal distribution assumption was confirmed using the Kolmogorov-Smirnov test and Anderson-Darling test. For normally distributed values, ordinary one-way ANOVA with Dunnett's multiple comparisons test was used for comparison of each group with the healthy group, and Pearson's correlation was performed to evaluate correlation between miRNA levels and clinical variables. For variables without normal distribution, Kruskal–Wallis test with Dunn's multiple comparisons test and Spearman's correlation were used. Receiver-operating characteristic (ROC) curves were performed to evaluate miRNAs as indicators of diseases. In the correlation test and ROC curve analysis, the log2-transformed FC values were used for relative miRNA expressions. Statistical analysis was performed by using the Prism 9 software (GraphPad Software, San Diego, CA, USA) and the SPSS 25.0 software (SPSS, Inc., Chicago, IL). A *p* < 0.05 indicated statistical significance. Unsupervised hierarchical clustering of miRNAs was conducted by MultiExperiment Viewer (MeV) software version 4.9.0.

## Results

### Classification by Disease Type

The classification by disease type included 73 dogs consisting of 10 healthy dogs, 35 dogs with MMVD, 21 dogs with PDA, and seven dogs with PS. In the MMVD group, one dog was in stage B1, seven dogs were in stage B2, 22 dogs were in stage C, and five dogs were in stage D based on the ACVIM consensus guideline. The clinical characteristics, breed distribution, and cardiovascular medication history of dogs included in this classification are shown in Table 1 and Supplementary Tables 2, 3, respectively.

**Table 1:** Clinical characteristics of dogs included in the classification by disease type.

**Variables**	**Healthy (*n* = 10)**	**MMVD (*n* = 35)**	**PDA (*n* = 21)**	**PS (*n* = 7)**	***p***
Age, years	2.55 ± 0.46	11.72 ± 2.43^a^	3.52 ± 2.40^b^	2.26 ± 1.85^b^	<0.0001
Male, *n* (%)	10 (100)	22 (63)	3 (14)	5 (71)	
BSA, m^2^	0.49 ± 0.02	0.26 ± 0.08^a^	0.24 ± 0.11^a^	0.33 ± 0.10	<0.0001
Heart rate, bpm	132 ± 12	151 ± 23	146 ± 19	131 ± 23	0.023
Systolic BP, mmHg	128 ± 8	142 ± 13^a^	134 ± 17	136 ± 11	0.014
Diastolic BP, mmHg	79 ± 4	96 ± 15^a^	87 ± 22	99 ± 10	0.005
Pulmonary hypertension, *n* (%)	0 (0)	25 (71)	3 (14)	7 (100)	
Mild	0	10	0	0	
Moderate	0	9	1	0	
Severe	0	6	2	7	
NTproBNP, pmol/L	439 ± 159	2,939 ± 2,611^a^	2,581 ± 3,379^a^	1,759 ± 1,583	<0.001
Echocardiography					
LA/Ao	1.18 ± 0.07	1.79 ± 0.47^a^	1.51 ± 0.34^a^	1.31 ± 0.05^b^	<0.0001
EDVI	79.63 ± 11.02	96.00 ± 45.93	130.00 ± 96.87	28.30 ± 15.64^b^, ^c^	0.001
ESVI	24.00 ± 4.22	13.92 ± 11.03	45.94 ± 51.63^b^	7.00 ± 7.92^a^, ^c^	<0.001
IVSdN	0.44 ± 0.07	0.45 ± 0.14	0.44 ± 0.13	0.60 ± 0.07^a^, ^b^, ^c^	0.011
LVIDdN	1.58 ± 0.10	1.63 ± 0.43	1.86 ± 0.56	1.02 ± 0.24^b^, ^c^	0.001
LVIDsN	0.94 ± 0.08	0.72 ± 0.25	1.12 ± 0.51^b^	0.54 ± 0.22^a^, ^c^	<0.001
RWT	0.49 ± 0.09	0.52 ± 0.18	0.48 ± 0.22	1.08 ± 0.43^a^, ^b^, ^c^	0.001
Symptomatic, *n*	0	27	21	7	
ACVIM stage, *n*					
B	NA	8	NA	NA	
C	NA	22	NA	NA	
D	NA	5	NA	NA	

*ACVIM, American College of Veterinary Internal Medicine; BP, blood pressure; BSA, body surface area; EDVI, end-diastolic volume index; ESVI, end-systolic volume index; IVSdN, normalized value of end-diastolic interventricular septal thickness; LA/Ao, ratio of left atrium to aorta; LVIDdN, normalized value of end-diastolic left ventricular internal dimension; LVIDsN, normalized value of end-systolic left ventricular internal dimension; MMVD, myxomatous mitral valve degeneration; NA, not applicable; NT-proBNP, N-terminal pro B-type natriuretic peptide; PDA, patent ductus arteriosus; PS, pulmonic stenosis; RWT, relative wall thickness*.

*Continuous variables were expressed as means ± standard deviations*.

a *p < 0.05 compared with the Healthy group*,

b *p < 0.05 compared with the MMVD group*,

c *p < 0.05 compared with the PDA group in the post-hoc comparison*.

#### Circulating miRNA Expression According to Disease Type

**Figure 2:**
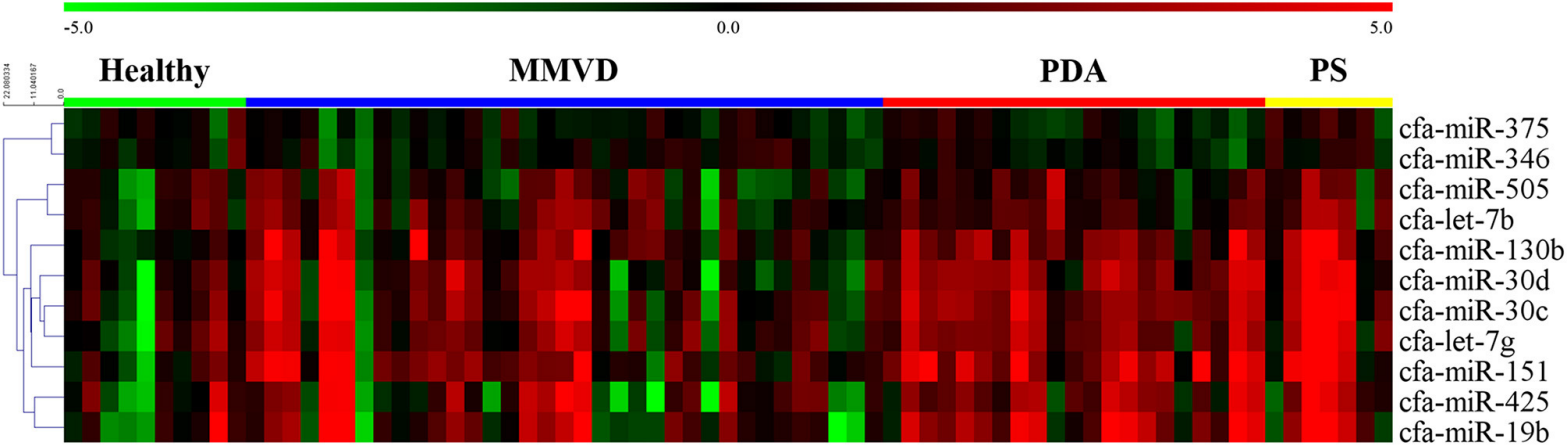
Heat map of overall miRNA expression according to disease type. Sample species shown at the top and the miRNA species shown on the right. Unsupervised average link hierarchical clustering of miRNAs with similar expressions using the Euclidean distance measure. Red indicates relatively high expression of miRNA, and green indicates low expression of miRNA. MMVD, myxomatous mitral valve degeneration; PDA, patent ductus arteriosus; PS, pulmonic stenosis.

The overall expression profiles of 11 miRNAs in each disease group and hierarchical clustering of miRNAs with similar expressions are shown in Figure 2.

**Figure 3:**
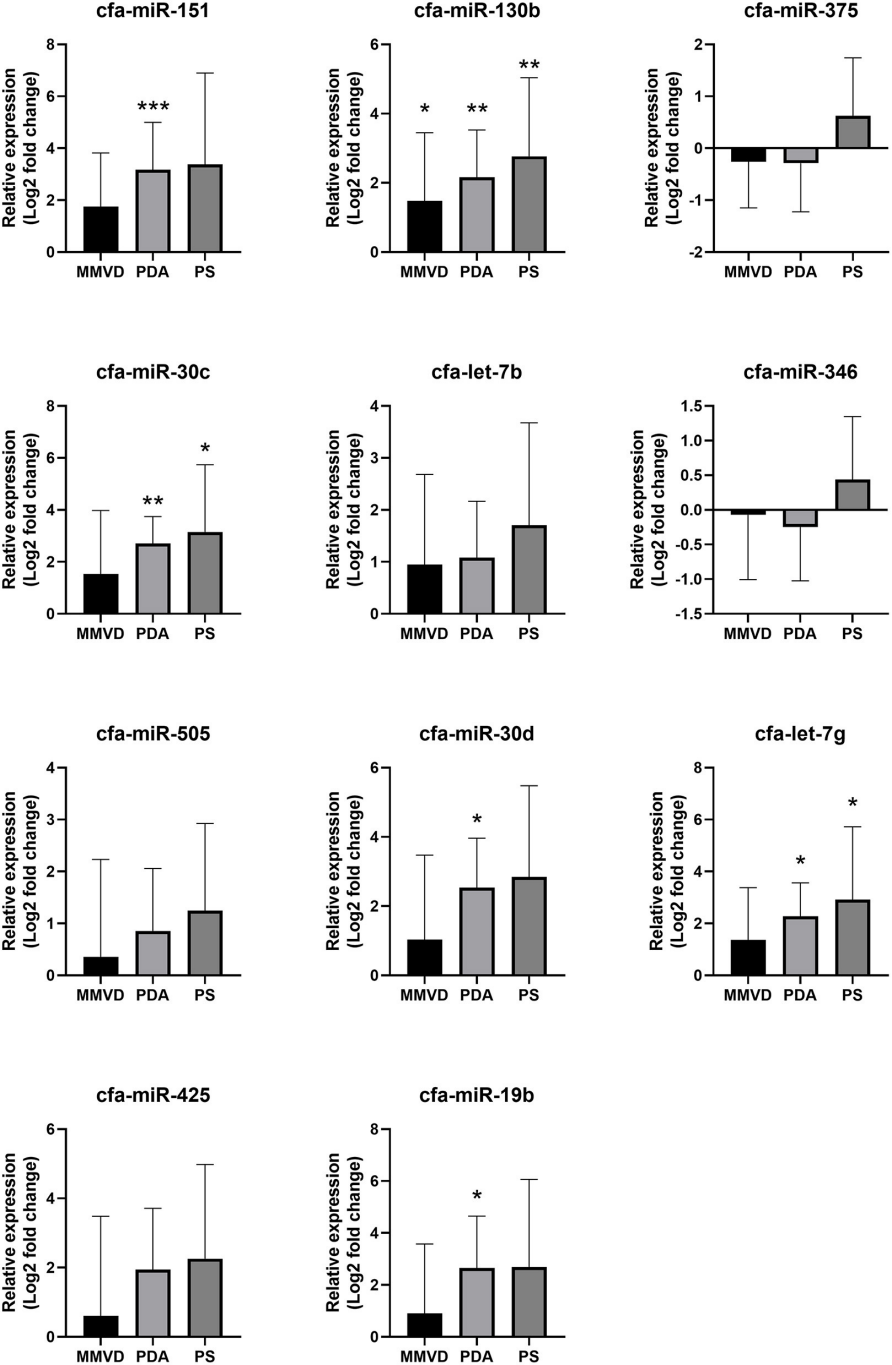
Relative expressions of circulating miRNAs according to disease type. Data were presented as log2 transformed fold change relative to the healthy group (mean and standard deviation). MMVD, myxomatous mitral valve degeneration; PDA, patent ductus arteriosus; PS, pulmonic stenosis. *p < 0.05, **p < 0.01, ***p < 0.001 in Kruskal–Wallis test, *p*-value adjusted by Dunn's multiple comparison test.

Compared with the healthy group, cfa-miR-151 was upregulated in the PDA group (FC = 9.00, *p* < 0.001); cfa-miR-30c was upregulated in the PDA group (FC = 6.53, *p* = 0.005) and PS group (FC = 8.88, *p* = 0.032); cfa-miR-130b was upregulated in the MMVD group (FC = 2.79, *p* = 0.047), PDA group (FC = 4.48, *p* = 0.001), and PS group (FC = 6.81, *p* = 0.009); cfa-let-7g was upregulated in the PDA group (FC = 4.83, *p* = 0.001) and PS group (FC = 7.55, *p* = 0.034); cfa-miR-30d was upregulated in the PDA group (FC = 5.81, *p* = 0.014); and cfa-miR-19b was upregulated in the PDA group (FC = 6.27, *p* = 0.018). The relative expressions of miRNAs in each group compared with the healthy group are shown in Figure 3.

Based on these results, cfa-miR-130b was selected as a candidate for promising common biomarker in various heart diseases.

#### Expression Profile of cfa-miR-130b in MMVD According to the ACVIM Stage

**Figure 4:**
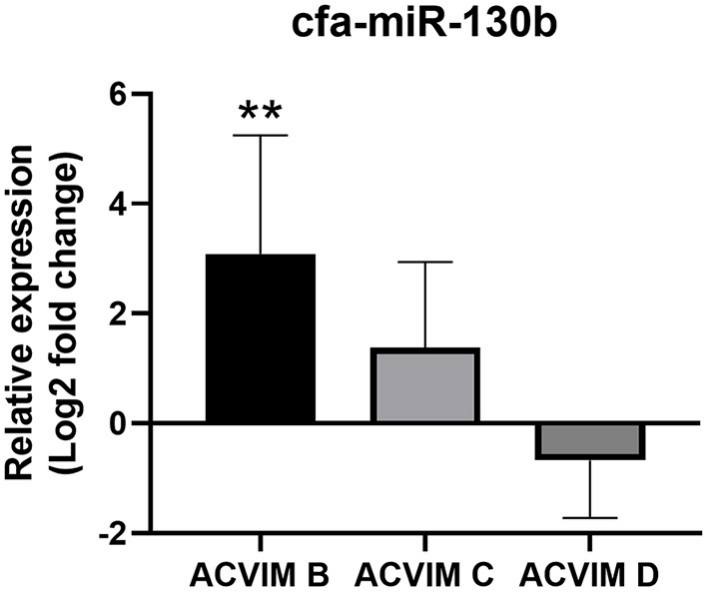
Relative expression of cfa-miR-130b in MMVD group according to ACVIM stage. Data were presented as log2 transformed fold change relative to the healthy group (mean and standard deviation). The cfa-miR-130b was significantly upregulated in stage B while no statistical difference from healthy group was observed in stage C and D. **p < 0.01, *p*-value adjusted by Dunn's multiple comparison test. Only dogs in the MMVD group were assigned the ACVIM stage in this study.

Although cfa-miR-130b was significantly upregulated in all three disease groups, some low expressions of cfa-miR-130b in MMVD group were observed in the heat map analysis (Figure 2). Therefore, cfa-miR-130b expression according to ACVIM stage was investigated to determine whether it changes according to the disease progression. As a result, cfa-miR-130b was significantly upregulated in stage B (*n* = 8) while no statistical difference from the healthy group was observed in stage C (*n* = 22) and stage D (*n* = 5) (Figure 4).

#### Correlation of cfa-miR-130b Level With Clinical Data

The correlation between cfa-miR-130b level and clinical data including medical records, echocardiographic results, and NT-proBNP level was investigated in groups that showed significant dysregulation of cfa-miR-130b compared with the healthy group (MMVD, MMVD stage B, PDA, and PS groups) (Supplementary Table 4). In the MMVD group, mild negative correlation between cfa-miR-130b and age (*r* = −0.352, *p* = 0.038) was observed. In the MMVD stage B group, cfa-miR-130b showed strong positive correlation (*r* > 0.7) with HR (*r* = 0.755, *p* = 0.031), NT-proBNP (*r* = 0.786, *p* = 0.021), and LA/Ao ratio (*r* = 0.719, *p* = 0.045).

#### ROC Analysis for cfa-miR-130b

**Figure 5:**
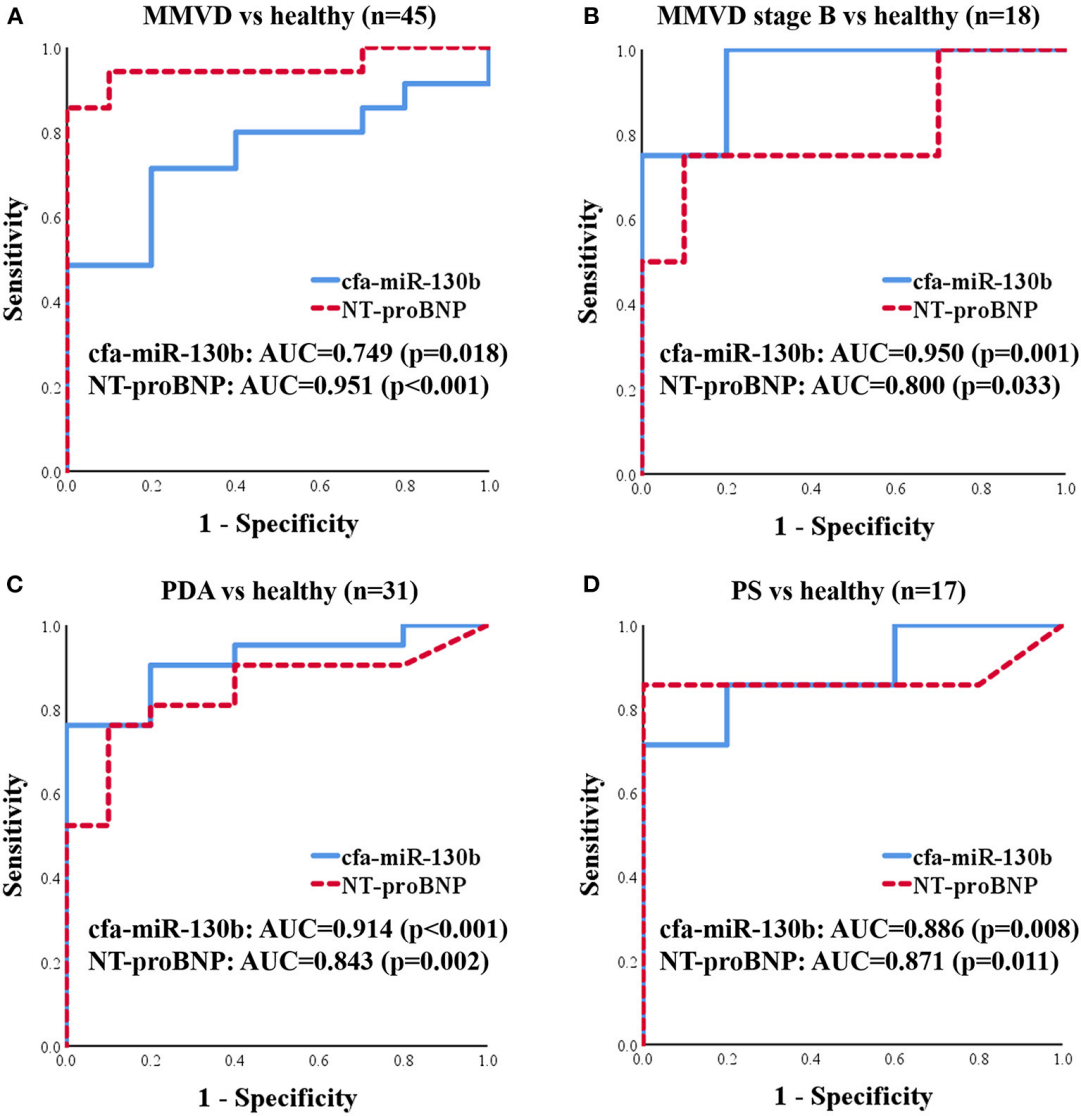
ROC curves for cfa-miR-130b and NT-proBNP to discriminate dogs with MMVD (*n* = 35) **(A)**, MMVD stage B (*n* = 8) **(B)**, PDA (*n* = 21) **(C)**, and PS (*n* = 7) **(D)** from healthy dogs (*n* = 10). AUC, area under curve; MMVD, myxomatous mitral valve degeneration; NT-proBNP, N-terminal pro B-type natriuretic peptide; PDA, patent ductus arteriosus; PS, pulmonic stenosis.

To evaluate cfa-miR-130b as a biomarker for various heart diseases, receiver operating characteristic (ROC) curves were analyzed in the groups, which showed significant dysregulation of cfa-miR-130b compared with the healthy group. Accordingly, ROC curves for cfa-miR-130b and NT-proBNP were generated to discriminate dogs with MMVD, MMVD stage B, PDA, and PS from healthy dogs (Figure 5). In all groups, cfa-miR-130b showed optimal area under the curve (AUC > 0.7). In MMVD stage B, PDA, and PS groups, cfa-miR-130b was more accurate than NT-proBNP for discriminating dogs with heart diseases from healthy dogs.

### Classification by Cardiac Hypertrophy Type

The classification by cardiac hypertrophy type included 78 dogs consisting of 10 healthy dogs, 53 dogs with eccentric cardiac hypertrophy, and 15 dogs with concentric cardiac hypertrophy. The clinical characteristics of dogs included in this classification are shown in Table 2.

**Table 2:** Clinical characteristics of dogs included in the classification by cardiac hypertrophy type.

**Variables**	**Healthy (*n* = 10)**	**Eccentric hypertrophy (*n* = 53)**	**Concentric hypertrophy (*n* = 15)**	***p***
Age, years	2.55 ± 0.46	9.00 ± 4.54^a^	6.49 ± 5.22	<0.001
Male, *n* (%)	10 (100)	24 (45)	8 (53)	
BSA, m^2^	0.49 ± 0.02	0.25 ± 0.09^a^	0.31 ± 0.12^a^	<0.0001
Heart rate, bpm	132 ± 12	149 ± 21	138 ± 33	0.056
Systolic BP, mmHg	128 ± 8	139 ± 14	135 ± 19	0.072
Diastolic BP, mmHg	79 ± 4	92 ± 16^a^	96 ± 16^a^	0.021
Pulmonary hypertension, *n* (%)	0 (0)	26 (49)	12 (80)	
Mild	0	10	2	
Moderate	0	10	0	
Severe	0	6	10	
NTproBNP, pmol/L	439 ± 159	2,836 ± 2,923^a^	2,216 ± 2,555	0.038
Echocardiography				
LA/Ao	1.18 ± 0.07	1.70 ± 0.44^a^	1.30 ± 0.15^b^	<0.0001
EDVI	79.63 ± 11.02	113.30 ± 71.96	34.09 ± 31.21^b^	<0.001
ESVI	24.00 ± 4.22	27.42 ± 36.80	8.63 ± 9.67	0.123
IVSdN	0.44 ± 0.07	0.43 ± 0.14	0.63 ± 0.11^a^, ^b^	<0.0001
LVPWdN	0.43 ± 0.07	0.43 ± 0.10	0.55 ± 0.15^a^, ^b^	<0.001
LVIDdN	1.58 ± 0.10	1.74 ± 0.49	1.13 ± 0.40^a^, ^b^	<0.0001
LVIDsN	0.94 ± 0.08	0.89 ± 0.41	0.57 ± 0.26^a^, ^b^	0.010
RWT	0.49 ± 0.09	0.49 ± 0.17	1.09 ± 0.50^a^, ^b^	<0.0001
Diagnosis	Healthy (*n* = 10)	MMVD (*n* = 35) PDA (*n* = 18)	PS (*n* = 7) MMVD-HAC (*n* = 4) CH-NCC (*n* = 3) TOF (*n* = 1)	

*BP, blood pressure; BSA, body surface area; CH-NCC, concentric hypertrophy by non-cardiac cause; EDVI, end-diastolic volume index; ESVI, end-systolic volume index; IVSdN, normalized value of end-diastolic interventricular septal thickness; LA/Ao, ratio of left atrium to aorta; LVIDdN, normalized value of end-diastolic left ventricular internal dimension; LVIDsN, normalized value of end-systolic left ventricular internal dimension; LVPWdN, normalized value of end-diastolic left ventricular free wall thickness; MMVD, myxomatous mitral valve degeneration; MMVD-HAC, myxomatous mitral valve degeneration with concurrent hyperadrenocorticism; NT-proBNP, N-terminal pro B-type natriuretic peptide; PDA, patent ductus arteriosus; PS, pulmonic stenosis; RWT; relative wall thickness; TOF, tetralogy of Fallot*.

*Continuous variables were expressed as means ± standard deviations*.

a *p < 0.05 compared with the Healthy group*,

b *p < 0.05 compared with the Eccentric hypertrophy group in the post-hoc comparison*.

#### Circulating miRNA Expression According to Cardiac Hypertrophy Type

**Figure 6:**
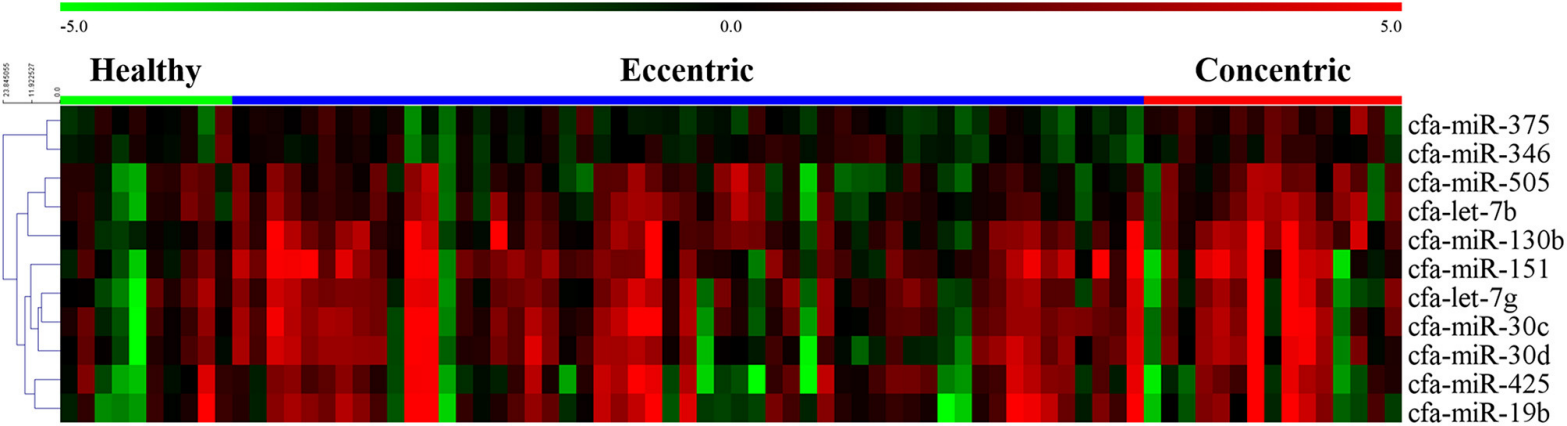
Heat map of overall miRNA expression according to cardiac hypertrophy type. Sample species shown at the top and the miRNA species shown on the right. Unsupervised average link hierarchical clustering of miRNAs with similar expressions using the Euclidean distance measure. Red indicates relatively high expression of miRNA, and green indicates low expression of miRNA.

The expression heat map and hierarchical clustering of 11 miRNAs according to cardiac hypertrophy type are shown in Figure 6.

**Figure 7:**
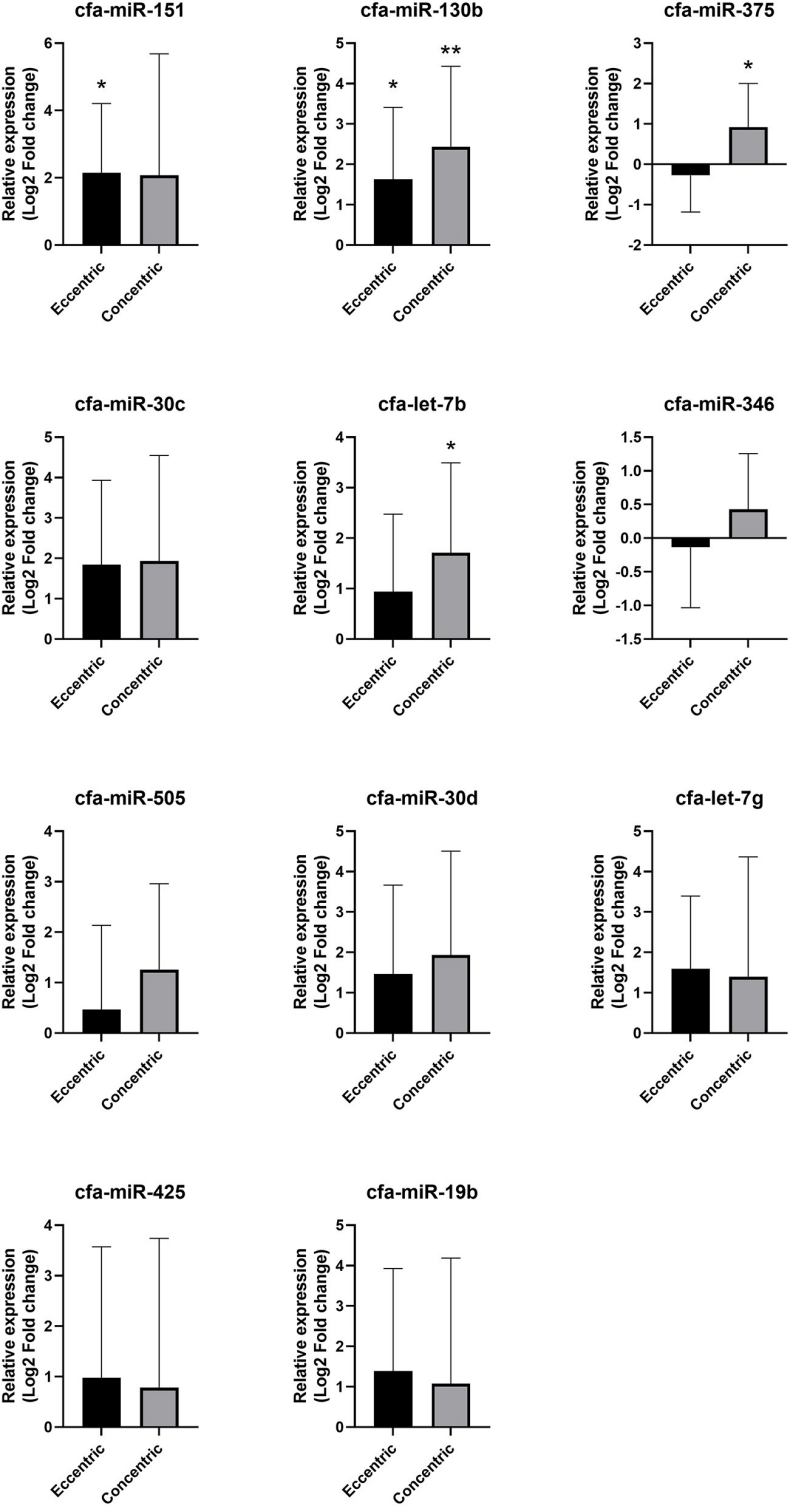
Relative expressions of circulating miRNAs according to cardiac hypertrophy type. Data were presented as log2 transformed fold change relative to the healthy group (mean and standard deviation). *p < 0.05, **p < 0.01 in one-way ANOVA among healthy, eccentric hypertrophy, and concentric hypertrophy groups, *p*-value adjusted by Dunnett's multiple comparison test.

Compared with the healthy group, cfa-miR-130b was upregulated in both the eccentric hypertrophy group (FC = 3.10, *p* = 0.014) and the concentric hypertrophy group (FC = 5.39, *p* = 0.002), cfa-miR-151 was upregulated in the eccentric hypertrophy group (FC = 4.44, *p* = 0.020), cfa-let-7b was upregulated in the concentric hypertrophy group (FC = 3.27, *p* = 0.020), and cfa-miR-375 was upregulated in the concentric hypertrophy group (FC = 1.90, *p* = 0.038). The relative expressions of miRNAs in each group compared with the healthy group are shown in Figure 7.

Based on these results, cfa-miR-375 and cfa-let-7b were selected for further analysis to identify specific association with concentric cardiac hypertrophy.

#### Correlation of cfa-miR-375 and cfa-let-7b Level With Clinical Data

The correlations between selected miRNAs and various clinical indices were analyzed in dogs with heart diseases (Supplementary Table 5). Significant positive correlations were observed between cfa-miR-375 and LV concentric hypertrophy indices, LVPWdN (*r* = 0.236, *p* = 0.046) and RWT (*r* = 0.290, *p* = 0.014).

#### ROC Analysis for cfa-miR-375 and cfa-let-7b

**Figure 8:**
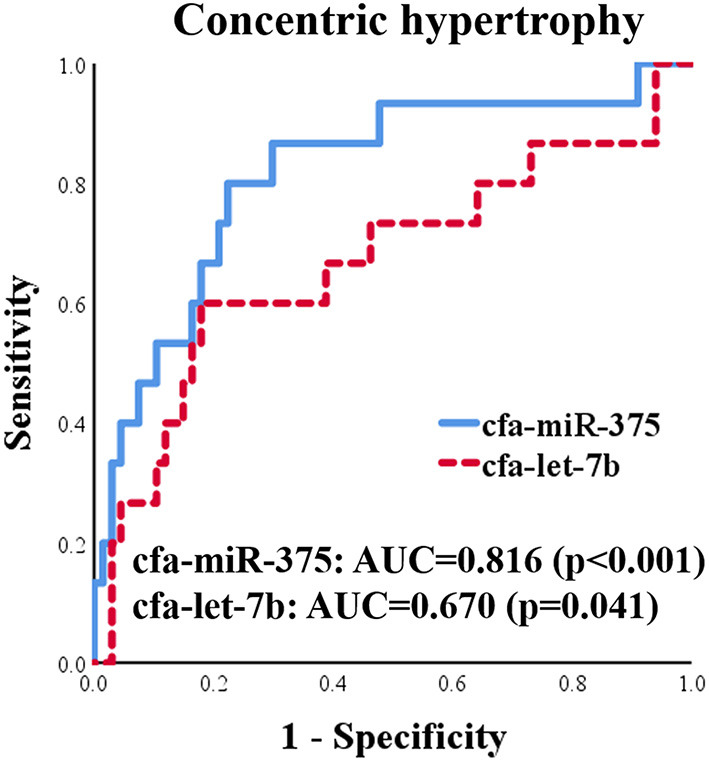
ROC curves for cfa-miR-375 and cfa-let-7b to distinguish dogs with concentric cardiac hypertrophy from dogs without concentric cardiac hypertrophy.

ROC curves for cfa-miR-375 and cfa-let-7b were generated to evaluate discriminatory power for distinguishing dogs with concentric cardiac hypertrophy from dogs without concentric cardiac hypertrophy, which included healthy and eccentric cardiac hypertrophy groups (Figure 8). Both cfa-miR-375 and cfa-let-7b were able to distinguish dogs with concentric hypertrophy from dogs without concentric hypertrophy. In particular, cfa-miR-375 was an accurate indicator associated with concentric cardiac hypertrophy (AUC = 0.816).

## Discussion

In this study, cfa-miR-130b showed several characteristics as a novel biomarker for various heart diseases. First, upregulation of cfa-miR-130b was detected in MMVD stage B, PDA, and PS groups, which indicates its wide range of applicability as a common biomarker in various heart diseases. Secondly, significant upregulation of cfa-miR-130b was observed in dogs with MMVD stage B, which indicates its suitability as an early diagnostic biomarker. Thirdly, the level of cfa-miR-130b was significantly correlated with HR, NT-proBNP, and LA/Ao in dogs with MMVD stage B, which shows that cfa-miR-130b may be able to represent cardiac status in dogs with early-stage MMVD. Fourthly, in ROC analysis, cfa-miR-130b showed higher sensitivity and specificity than NT-proBNP in MMVD stage B, PDA, and PS groups. However, since no significant changes of cfa-miR-130b level were observed in advanced stages of MMVD (stage C and D), NT-proBNP was a more accurate biomarker than cfa-miR-130b when analyzed in the entire MMVD group. Based on these results, cfa-miR-130b is considered to be a useful biomarker for early detection and monitoring of various heart diseases, and may be more valuable when used in combination with NT-proBNP.

Since cfa-miR-130b was commonly upregulated in various heart diseases regardless of the type of disease or type of cardiac hypertrophy, it may be related to common physiological or pathological changes that can be induced by various heart diseases. In addition, significant upregulation of cfa-miR-130b in MMVD stage B, an early asymptomatic disease state, suggests that the expression of cfa-miR-130b may be associated with early gene response to cardiac stress, which is required for subsequent cardiac hypertrophy, fibrosis, and eventual development of heart failure (29). However, the specific target genes and pathways of cfa-miR-130b were not identified in this study, which remains to be clarified in future studies in dogs.

In a previous study in rats with induced myocardial infarction (30), peroxisome proliferator-activated receptor γ (PPAR-γ) was verified to be the target of miR-130b, and expression of miR-130b was associated with NFκB-mediated cardiac inflammation and TGF-β1-mediated cardiac fibrosis. Similar to this result, a prior study in humans reported that circulating miR-130b regulated expression of PPAR-γ and was related to development of coronary artery disease (31). Meanwhile, miR-130b was also reported to be upregulated in response to hypoxic condition and regulated target gene DDX6 to increase hypoxia-inducible factor 1-alpha, which is a key factor in protection against myocardial ischemic injury (32, 33). Despite the fact that most miRNAs and their targets are known to be highly conserved across different species, conserved miRNAs can exhibit different expression levels or target regulation in different species (34). In addition, it is known that one miRNA can regulate several target genes and have various functions in different conditions (35). Therefore, the target genes and specific pathways of cfa-miR-130b in heart diseases should be identified and validated in further studies in dogs.

In the present study, the upregulation of cfa-miR-130b level was observed only in the MMVD stage B group. Therefore, cfa-miR-130b is considered not to be a reliable biomarker for the entire MMVD group. Similarly, in a previous study in dogs (13), cfa-miR-30b was significantly dysregulated only in MMVD stage B dogs when compared with healthy dogs, although the reason was not discussed in that study. This was an interesting finding, but the reason for the significant dysregulation only in MMVD stage B group is difficult to clarify in this study. The largely different medication history between stage B vs. stage C and D (Supplementary Table 3) may be considered as one of the possible reasons. Further controlled studies are expected to clarify and explain the findings of this study.

Meanwhile, although cfa-miR-375 and cfa-let-7b were significantly associated with concentric cardiac hypertrophy, cfa-let-7b alone seems to be less specific for concentric cardiac hypertrophy than cfa-miR-375. In ROC and correlation analysis, the specificity of cfa-let-7b was lower than that of cfa-miR-375, and no correlation was observed between cfa-let-7b and concentric cardiac hypertrophy indices. In addition, in a previous study (11), cfa-let-7b was upregulated in dogs with MMVD. Therefore, based on the results of the previous and present studies (11), cfa-let-7b is thought to be associated with both eccentric and concentric cardiac hypertrophy in dogs. The reason why significant dysregulation of cfa-let-7b was not observed in MMVD or eccentric hypertrophy group in the present study may be the relatively low sensitivity of cfa-let-7b in heart diseases or the insufficient number of samples in this study.

A previous study in mice reported that let-7b was upregulated by thioredoxin 1 (Trx1) in angiotensin-II (AT-II)-induced cardiac hypertrophy and inhibited AT-II by targeting cyclin D2 (36). The upregulation of Trx1 and downregulation of angiotensinogen, which is a precursor of AT-II, were also reported in mitral valves of MMVD dogs, suggesting that there may be a similar pathway involving cfa-let-7b (11). However, the direct target genes and pathways of cfa-let-7b are not identified in dogs yet.

In contrast to cfa-let-7b, cfa-miR-375 seems to be more specifically related to concentric cardiac hypertrophy based on the results of ROC and correlation analysis in this study. In previous human studies, miR-375 was upregulated in patients with concentric cardiac hypertrophy (37) and downregulated in patients with eccentric cardiac hypertrophy (heart failure with reduced ejection fraction) (38). These expression patterns of miR-375 from human studies are similar to those observed in cfa-miR-375 in this study, although no statistical significance was observed in the eccentric cardiac hypertrophy group. Further research is required to verify the expression and specific role of cfa-miR-375 in dogs with different types of cardiac hypertrophy.

In previous studies in mice (39, 40), inhibition of miR-375 by interleukin-10 administration or anti-miR-375 therapy enhanced cardiac recovery and reduced inflammatory response after myocardial infarction by activation of the PDK-1-AKT pathway. In another study (41), upregulation of miR-375 in P19 cell resulted in inhibition of cell proliferation, increased cell apoptosis, and disruption of cardiomyocyte differentiation through Notch signaling pathway. On the other hand, a contrasting result was also reported in a prior study in humans (42), in which downregulation of miR-375 was observed in myocardial infarction patients and overexpression of miR-375 by mimic-miR-375 prevented hypoxia/reoxygenation-induced cardiac injury by upregulating Nemo-like kinase (NLK) gene.

This study has several limitations. First, the age, breed, sex, and BSA of the healthy dogs could not be matched with the dogs with heart diseases. In this study, only young male beagle dogs were included in the healthy group because those samples were the only available samples that were definitely confirmed to be healthy in our sample archive. Since miRNAs are known to be associated with various physical conditions and diseases (43), and very little is known in dogs, we tried to use samples from definitely healthy dogs without any disease. In previous studies in humans (44–48), several miRNAs have been reported to be associated with age, sex, and racial difference. However, the relationship between those factors and the miRNAs investigated in this study has not been identified in dogs. In this study, negative correlation between age and cfa-miR-130b was observed in the MMVD group. This finding could not be confirmed in healthy dogs because only young dogs were included in the healthy group. In addition, it is difficult to elucidate the cause of this correlation because factors that could affect miRNA expression in the MMVD group such as severity of disease and use of medication were not controlled in this study. Regarding the sex, there were no significant differences between male and female in MMVD stage B, PDA, and PS groups in which cfa-miR-130b showed a significant upregulation (data not shown). However, this also should be investigated in healthy dogs. Therefore, further controlled studies are needed to clarify the association between miRNA expression and age, as well as breed, sex, and BSA.

Secondly, since only one dog with MMVD was in stage B1, the data between stage B1 and B2 could not be compared properly. If the miRNA expressions can differentiate dogs with stage B2 from B1, it will be beneficial for both early diagnosis and treatment of MMVD in dogs. In addition, seven dogs were included in the PS group in this study, which was a relatively small number compared with other groups. Further large-scale studies are expected in the future. Moreover, in order to verify the target genes and potential mechanisms of the miRNAs identified in this study, and to apply them as therapeutic agents, further follow-up studies with miRNA mimics or miRNA inhibitors (anti-miRs) are necessary in dogs with heart diseases (6).

To our knowledge, this is the first study to investigate circulating miRNAs in dogs with various heart diseases to identify and characterize them as novel biomarkers and possible therapeutic targets, according to the disease type and cardiac hypertrophy type. The miRNAs identified in this study may be used as promising novel biomarkers and candidates for therapeutic targets in various canine heart diseases. The results of this study are expected to provide basis for further studies and accelerate the application of new diagnostic and therapeutic approaches for dogs with heart diseases.

## Data Availability Statement

The original contributions presented in the study are included in the article/Supplementary Material, further inquiries can be directed to the corresponding author.

## Ethics Statement

Ethical review and approval was not required for the animal study because informed consent was obtained from the owner for sample collection of client-owned dog. Stored serum samples of healthy dogs from a previous study were utilized in this study. The previous study was conducted under the supervision of Korea Institute for Advancement of Technology (KIAT) for Regional Specialized Industry Development Program (R&D, R0006046) and approved by KBNP Institutional Animal Care and Use Committee (KBNP 18-01-01).

## Author Contributions

W-BR, M-HK, and H-MP conceived and designed the study. W-BR, D-WS, H-SK, and G-WL participated in sample and data collection. W-BR and D-WS curated the data and carried out the research. W-BR, M-HK, D-WS, and H-MP analyzed the data. W-BR wrote the manuscript. All authors have read and approved the final manuscript.

## Conflict of Interest

The authors declare that the research was conducted in the absence of any commercial or financial relationships that could be construed as a potential conflict of interest.

## Publisher's Note

All claims expressed in this article are solely those of the authors and do not necessarily represent those of their affiliated organizations, or those of the publisher, the editors and the reviewers. Any product that may be evaluated in this article, or claim that may be made by its manufacturer, is not guaranteed or endorsed by the publisher.
