# Supplementary material for: Identification and Characterization of Circulating MicroRNAs as Novel Biomarkers in Dogs With Heart Diseases
Source: Front Vet Sci. 2021 Oct 11;8:729929. doi: 10.3389/fvets.2021.729929 (PMC8542680; doi:10.3389/fvets.2021.729929)
Supplement: Supplementary file 1 [file Data_Sheet_1.docx]

Supplementary Material

# Supplementary Table

**Supplementary Table 1.**The sequences of miRNAs and accession numbers of the primers used in the study

| Primer | Target sequence | Sequence Length | Accession |
| --- | --- | --- | --- |
| Cf_miR-30c_1 miScript Primer Assay | UGUAAACAUCCUACACUCUCAGCU | 24 | MIMAT0006605 |
| Cf_let-7b_1 miScript Primer Assay | UGAGGUAGUAGGUUGUGUGGUU | 22 | MIMAT0009836 |
| Cf_miR-346_1 miScript Primer Assay | UGUCUGCCCGCAUGCCUGCCUCU | 23 | MIMAT0009898 |
| Cf_miR-505_1 miScript Primer Assay | GGGAGCCAGGAAGUAUUGAUGU | 22 | MIMAT0009908 |
| Cf_miR-30d_1 miScript Primer Assay | UGUAAACAUCCCCGACUGGAAGCU | 24 | MIMAT0006616 |
| Cf_let-7g_1 miScript Primer Assay | UGAGGUAGUAGUUUGUACAGUU | 22 | MIMAT0006637 |
| Cf_miR-425_1 miScript Primer Assay | AAUGACACGAUCACUCCCGUUGA | 23 | MIMAT0006639 |
| Cf_miR-19b_1 miScript Primer Assay | UGUGCAAAUCCAUGCAAAACUG | 22 | MIMAT0006652 |
| Cf_miR-151_1 miScript Primer Assay | UCGAGGAGCUCACAGUCUAGU | 21 | MIMAT0006615 |
| Cf_miR-130b_1 miScript Primer Assay | CAGUGCAAUGAUGAAAGGGCAU | 22 | MIMAT0006659 |
| Cf_miR-375_1 miScript Primer Assay | UUUGUUCGUUCGGCUCGCGUGA | 22 | MIMAT0009871 |

Primers were designed and provided by commercial laboratory (Qiagen, Germany).

**Supplementary Table 2.**Breed distribution of dogs included in the classification by disease type

| **Breed, n** | **Healthy** | **MMVD** | **PDA** | **PS** | **Total** |
| --- | --- | --- | --- | --- | --- |
|  | **(n=10)** | **(n=35)** | **(n=21)** | **(n=7)** | **(n=73)** |
| Beagle | 10 | 1 |  |  | 11 |
| Maltese |  | 18 | 12 |  | 30 |
| Shih Tzu |  | 9 |  |  | 9 |
| Pomeranian |  | 3 | 5 | 3 | 11 |
| Mixed |  | 1 | 1 | 1 | 3 |
| Pekingese |  | 1 |  |  | 1 |
| Poodle |  | 1 |  | 1 | 2 |
| Yorkshire Terrier | | 1 |  |  | 1 |
| Bichon Frise |  |  | 1 |  | 1 |
| Cocker Spaniel |  |  | 1 |  | 1 |
| Welshi Corgi |  |  | 1 |  | 1 |
| Chihuahua |  |  |  | 1 | 1 |
| French Bulldog |  |  |  | 1 | 1 |

MMVD, myxomatous mitral valve degeneration; PDA, patent ductus arteriosus; PS, pulmonic stenosis.

**Supplementary Table 3.** Cardiovascular medication history of dogs included in the classification by disease type

| **Variables** | **MMVD** | | | **PDA** | **PS** |
| --- | --- | --- | --- | --- | --- |
|  | **ACVIM class** | | |  |  |
|  | **B** | **C** | **D** |  |  |
|  | **(n=8)** | **(n=22)** | **(n=5)** | **(n=21)** | **(n=7)** |
| Drugs treated, n (%) |  |  |  |  |  |
| Pimobendan | 2 (25) | 13 (59.1) | 5 (100) | 14 (66.7) | 0 (0) |
| Furosemide | 6 (75) | 20 (90.9) | 2 (40) | 18 (85.7) | 5 (71.4) |
| Torsemide | 0 (0) | 0 (0) | 3 (60) | 1 (4.8) | 0 (0) |
| Ramipril | 6 (75) | 15 (68.2) | 5 (100) | 16 (76.2) | 4 (57.1) |
| Enalapril | 0 (0) | 2 (9.1) | 0 (0) | 2 (9.5) | 0 (0) |
| Amlodipine | 0 (0) | 4 (18.2) | 0 (0) | 1 (4.8) | 0 (0) |
| Spironolactone | 5 (62.5) | 11 (50) | 4 (80) | 7 (33.3) | 3 (42.9) |
| Digoxin | 0 (0) | 1 (4.5) | 3 (60) | 0 (0) | 0 (0) |
| Sildenafil | 4 (50) | 11 (50) | 4 (80) | 7 (33.3) | 6 (85.7) |
| Beraprost | 0 (0) | 0 (0) | 0 (0) | 1 (4.8) | 0 (0) |
| Atenolol | 0 (0) | 1 (4.5) | 0 (0) | 0 (0) | 6 (85.7) |
| Diltiazem | 0 (0) | 0 (0) | 0 (0) | 0 (0) | 0 (0) |
| Hydralazine | 0 (0) | 1 (4.5) | 3 (60) | 1 (4.8) | 0 (0) |
| Pentoxifylline | 0 (0) | 4 (18.2) | 0 (0) | 1 (4.8) | 4 (57.1) |

ACVIM, American College of Veterinary Internal Medicine; MMVD, myxomatous mitral valve degeneration; PDA, patent ductus arteriosus; PS, pulmonic stenosis.

**Supplementary Table 4.** Correlation between cfa-miR-130b and the clinical data

| Variables | MMVD | | MMVD stage B | | PDA | | PS | |
| --- | --- | --- | --- | --- | --- | --- | --- | --- |
|  | r | p | r | p | r | p | r | p |
| Age | **-0.352*** | **0.038** | 0.307 | 0.460 | 0.087 | 0.708 | -0.234 | 0.613 |
| BSA | 0.267 | 0.121 | -0.048 | 0.911 | 0.100 | 0.666 | 0.214 | 0.645 |
| HR | -0.093 | 0.596 | **0.755*** | **0.031** | -0.028 | 0.903 | 0.000 | 1.000 |
| SBP | -0.263 | 0.127 | 0.347 | 0.399 | 0.184 | 0.439 | 0.750 | 0.052 |
| DBP | -0.036 | 0.839 | -0.048 | 0.911 | 0.256 | 0.276 | -0.143 | 0.760 |
| NT-proBNP | -0.152 | 0.385 | **0.786*** | **0.021** | -0.045 | 0.846 | -0.536 | 0.215 |
| FS | 0.019 | 0.915 | 0.476 | 0.233 | 0.146 | 0.528 | 0.464 | 0.294 |
| EF | -0.004 | 0.981 | 0.476 | 0.233 | 0.126 | 0.587 | 0.464 | 0.294 |
| LA/Ao | -0.040 | 0.824 | **0.719*** | **0.045** | -0.244 | 0.301 | 0.174 | 0.742 |
| S’ | 0.018 | 0.918 | 0.643 | 0.086 | 0.030 | 0.899 | 0.286 | 0.535 |
| E’/A’ | 0.184 | 0.298 | -0.476 | 0.233 | -0.335 | 0.149 | -0.393 | 0.383 |
| E/E’ | -0.190 | 0.281 | 0.286 | 0.493 | -0.281 | 0.229 | 0.143 | 0.760 |
| IVSdN | -0.038 | 0.829 | -0.143 | 0.736 | 0.253 | 0.268 | 0.036 | 0.939 |
| LVPWdN | -0.025 | 0.889 | -0.554 | 0.154 | 0.182 | 0.429 | 0.393 | 0.383 |
| RWT | -0.004 | 0.981 | -0.476 | 0.233 | 0.432 | 0.051 | -0.036 | 0.939 |
| LVMI | -0.127 | 0.466 | -0.024 | 0.955 | -0.271 | 0.234 | 0.393 | 0.383 |
| LVIDdN | -0.120 | 0.492 | 0.548 | 0.160 | -0.423 | 0.056 | 0.071 | 0.879 |
| LVIDsN | -0.093 | 0.594 | -0.167 | 0.693 | -0.324 | 0.152 | -0.036 | 0.939 |

Values are correlation coefficient (r) and p-value. Log2 transformed fold change values were used for relative expression of miRNA level.

BSA, body surface area; DBP, diastolic blood pressure; E/E’, ratio of the peak velocity of the early diastolic trans-mitral flow to that of the early diastolic mitral annular motion; EF, ejection fraction; E’/A’, ratio of the peak velocity of the early diastolic mitral annular motion to that of the late diastolic mitral annular motion; FS, fractional shortening; HR, heart rate; IVSdN; normalized value of end-diastolic interventricular septal thickness; LA/Ao, ratio of left atrium to aorta; LVIDdN, normalized value of end-diastolic left ventricular internal dimension; LVIDsN, normalized value of end-systolic left ventricular internal dimension; LVMI, left ventricular mass index; LVPWdN, normalized value of end-diastolic left ventricular free wall thickness; NT-proBNP, N-terminal pro B-type natriuretic peptide; RWT, relative wall thickness; SBP, systolic blood pressure; S’, the peak velocity of the systolic mitral annular motion.

Bold text indicates significant correlation (*p<0.05).

**Supplementary Table 5.** Correlation between cfa-miR-375, cfa-let-7b and the clinical data in dogs with heart diseases

| Variables | cfa-miR-375 | | cfa-let-7b | |
| --- | --- | --- | --- | --- |
|  | r | p | r | p |
| Age | 0.059 | 0.622 | -0.228 | 0.054 |
| BSA | 0.020 | 0.870 | 0.161 | 0.177 |
| HR | -0.113 | 0.346 | -0.171 | 0.151 |
| SBP | -0.169 | 0.162 | -0.176 | 0.145 |
| DBP | -0.012 | 0.919 | -0.104 | 0.394 |
| NT-proBNP | 0.147 | 0.222 | -0.117 | 0.332 |
| FS | -0.037 | 0.761 | 0.032 | 0.791 |
| EF | -0.002 | 0.984 | 0.053 | 0.662 |
| LA/Ao | -0.188 | 0.121 | -0.168 | 0.168 |
| S’ | -0.028 | 0.819 | -0.041 | 0.739 |
| E’/A’ | -0.185 | 0.127 | 0.082 | 0.505 |
| E/E’ | 0.049 | 0.688 | -0.206 | 0.089 |
| IVSdN | 0.224 | 0.059 | 0.043 | 0.721 |
| LVPWdN | **0.236*** | **0.046** | 0.050 | 0.674 |
| RWT | **0.290*** | **0.014** | 0.026 | 0.827 |
| LVMI | 0.013 | 0.916 | -0.103 | 0.389 |
| LVIDdN | -0.184 | 0.122 | -0.161 | 0.176 |
| LVIDsN | -0.096 | 0.422 | -0.149 | 0.213 |

Values are correlation coefficient (r) and p-value. Log2 transformed fold change values were used for relative expression of miRNA level.

BSA, body surface area; DBP, diastolic blood pressure; E/E’, ratio of the peak velocity of the early diastolic trans-mitral flow to that of the early diastolic mitral annular motion; EF, ejection fraction; E’/A’, ratio of the peak velocity of the early diastolic mitral annular motion to that of the late diastolic mitral annular motion; FS, fractional shortening; HR, heart rate; IVSdN; normalized value of end-diastolic interventricular septal thickness; LA/Ao, ratio of left atrium to aorta; LVIDdN, normalized value of end-diastolic left ventricular internal dimension; LVIDsN, normalized value of end-systolic left ventricular internal dimension; LVMI, left ventricular mass index; LVPWdN, normalized value of end-diastolic left ventricular free wall thickness; NT-proBNP, N-terminal pro B-type natriuretic peptide; RWT, relative wall thickness; SBP, systolic blood pressure; S’, the peak velocity of the systolic mitral annular motion.

Bold text indicates significant correlation (*p<0.05).
